# Code-switching costs from Chinese-English relative clauses processing

**DOI:** 10.3389/fpsyg.2023.1144530

**Published:** 2023-05-25

**Authors:** Wanying Hu, Yang Zhao

**Affiliations:** ^1^College of International Education, Minzu University of China, Beijing, China; ^2^School of Chinese as a Second Language, Peking University, Beijing, China

**Keywords:** code-switching cost, Chinese-English bilingual, relative clause, syntactic processing, bilingualism

## Abstract

**Introduction:**

The source of costs is a primary concern in code-switching, yet a consensus has not yet been reached. This study investigates whether code-switching during syntactic processing in Chinese-English dual languages results in a cost.

**Methods:**

We use Chinese and English relative clauses in either object (Experiment 1) or subject (Experiment 2, which has a more complex structure) positions to test the costs in syntactic processing. Forty-seven Chinese-English bilinguals and 17 English-Chinese bilinguals participated in acceptability judgment tests and self-paced reading experiments.

**Results:**

The statistical findings indicate that syntactic processing is a source of the costs incurred in code-switching, as evidenced by the code-switching costs observed in the head movement during relative clause comprehension.

**Discussion:**

The outcomes are consistent with the implications of the 4-Morpheme Model and the Matrix Language Framework. Additionally, the experiment shows that the processing of relative clauses depends on the underlying structures, which is consistent with the Dependency Locality Theory.

## 1. Introduction

Code-switching, shortened as CS, refers to the alternating use of two languages in a single utterance, a sentence, or other language components, which is one of the prominent features of bilingual language life (Toribio and Bullock, 2009). Within the field of psycholinguistic research on code-switching, the switching cost is a widely discussed phenomenon that refers to the cognitive consumption required by bilingual individuals when transitioning from one language to another, resulting in longer response times, higher error rates, and higher consumption of cognitive resources. This cost is quantifiable, easy to measure, and comparable, which reflects the language control ability of bilinguals (Zhang et al., 2020). Most research has found costs in code-switching understanding and producing with the discussions on influencing factors and sources of the costs.

There is currently no consensus on whether the cost of code-switching comes solely from task switching or also from language processing. There are discussions primarily on the lexical level and little research on the sentence level. This study provides evidence for the mechanism of code-switching costs at the syntactic level by examining whether code-switching will occur during the code-switching in the head movement of relative clauses comprehension in two experiments based on the different word orders of Chinese and English relative clauses. Experiment 1 examines the cost in object position relative clauses. While Experiment 2 involves the modification of materials to incorporate subject position relative clauses within an indirect speech context, thereby eliminating the potential influence of sentence-ending effects and the ambiguity associated with the definition of the matrix language.

### 1.1. The sources of code-switching costs

The cost of code-switching remains a topic of ongoing debate with questions surrounding its origin and whether language processing serves as the underlying reason or not. Currently there are two main explanations. The first proposes that only non-verbal processing is accountable for code-switching costs (Thomas and Allport, 2000; Zhang and Wang, 2012; Bosma and Pablos, 2020). While some studies contend that the cost of code-switching is also influenced by language processing, particularly bilingual mental lexicon processing (Grainger and Beauvillain, 1987; Casaponsa et al., 2020).

The first view is based on the idea that the code-switching process is controlled by a general cognitive control mechanism. Some studies have found that code-switching cost equals task-switching cost, which refers the increased response times and error rates that occur when individuals switch from one cognitive task to another (Monsell, 2003; Huang and Lin, 2010). Since code-switching and non-code-switching belong to two types of tasks with different difficulties using different strategies (Dijkstra and van Heuven, 2002), code-switching could be an inherent process of task-switching and entirely unrelated to language processing. Task-switching costs have been found in many code-switching studies too (Thomas and Allport, 2000; Cui and Zhang, 2010; Yim and Bialystok, 2012; Zhang and Wang, 2012; Liu et al., 2015; Timmermeister et al., 2020).

Liu et al. (2015) used alpha-number classification and picture-naming to investigate the relationship between code-switching and task-switching. Simple and mixed conditions were presented as separate blocks. In the alpha-number classification task, the alpha-number combination’s position was stationary in the simple condition. The task was to determine whether a letter was a vowel or a consonant. Under the mixed condition, the alpha-number pair’s position was not stationary. The task was to determine whether the letter was a vowel or consonant or to judge the parity of the number based on where the alpha-number combination appeared. The mixed conditions tested task-switching costs. The picture-naming analyzed the code-switching costs with similar sets. The simple condition required the subjects to use their mother tongue. In the mixed condition, they used either their mother tongue or second language depending on the position. In regard to the findings, there was no statistically significant interaction observed between task types and task conditions (simple/mixed). This suggests that the performance patterns of code-switching and task-switching costs are similar. According this study, it can be inferred that task-switching is responsible for all the costs associated with code-switching. However, Liu et al. (2015) concentrated on the output process of Cantonese-Putonghua bidialectal switching, which took place within the same language system and thus did not reflect the switching of two distinct languages. Switching between these two dialects may differ from switching between two distinct languages. Furthermore, their research, as well as several other experiments (Zhang and Wang, 2012), employed materials from the lexical level, which did not include switching in syntactic processing. No evidence is provided for whether there are costs from syntactic processing.

To the second view, certain studies have indicated that the cost of code-switching may arise from processing in the mental lexicon. As per this view, two distinct languages are stored in separate mental lexicons. When bilingual individuals process the orthographic or phonological information in one language, they activate only the corresponding mental lexicon. When code-switching occurs, it requires activation of another mental lexicon, leading to switching costs due to increased cognitive resource consumption. Grainger and Beauvillain (1987) has proved that there is a bilingual competition in sub-word processing by observing orthographic processing, and showed that the code-switching cost did occur at this stage.

The researchers categorized the words into two groups based on the independent variable of whether the targets were consistent with the orthographies of both languages or just one language, as demonstrated in Example (1):

1. A.Consistent with both English-French orthographies: brain (En), Canot (boat, Fr).
  B.Consistent with English or French orthographies: white (En), proie (spoils of war, Fr).

In group A, the word “brain” is an English word, and its letter combination is also acceptable in French, and “canot” too. In group B, the “wh” in “white” is a letter combination that is allowed in English but not in French, so the word does not fit in both English-French orthographies, as does “proie.” In the word-nonword judgment task, there was a significant switching cost in group B but not in group A. Grainger and Beauvillain (1987) suggested that this phenomenon is related to the language-specific orthography in the mental lexicon, wherein different orthographies from different languages are stored separately and not activated concurrently. The change of orthographies leads to code-switching cost, indicating that the orthographic processing stage is the source of code-switching cost: in cases where a word possesses language-specific orthographic features, such as the English word “white” with the letter combination “wh,” the decoding process is limited to activating only the orthography that is consistent with the word (other English words), while the orthography that is inconsistent with the word (i.e., French words) is not activated. When switching to the French word, bilinguals need to activate French orthography, resulting in costs. If a word’s orthography is consistent with that of both languages, it activates dual language orthographies. Subsequently switching to French does not necessitate reactivation of the orthography, thereby reducing consumption.

The main focus of recent research has been on whether the costs of code-switching originate from the mental lexicon or simply from task-switching as a general cognitive process. Syntactic processing is rarely discussed. Myers-Scotton (1993, 2002) proposed that the Matrix Language Framework (shortened as MLF) and later the 4-M model, dividing code-switching into two phases: mental lexicon and formulator. The MLF puts forth the Uniform Structure Principle and the Differential Access Hypothesis. The former claims that when only one lexeme in a mixed language originates from the embedded language, the order should be consistent with that of the matrix language. The differential access Hypothesis states that no natural code-switching occurs in the formulator. This assertion implies that the source of the cost is the formulator. Li et al. (2018) use eye movement technology and discover switching costs in language processing. Dijkstra and van Heuven (2002) also argues that the code-switching cost may have multiple sources. Language processing and task switching are both potential sources of code-switching cost. The verification of syntactic processing costs, however, is still inadequate at this time.

### 1.2. Research on relative clauses processing

The relative clause is one of the most studied structural phenomena in language research. As the Chinese relative clause has a non-dominant word order (SVO language with a Relative-N order), the study of the relative clause holds typological significance. Research conducted on English and several other Indo-European languages has demonstrated that subject clauses are acquired prior to object clauses in both children and adults, and possess advantages in processing (Liu et al., 2011; Gutierrez-Mangado and Ezeizabarrena, 2012). Many theories, including Dependency Locality Theory, Relativized Minimality, Accessibility Theory, and Perspective Conversion Theory, have been proposed to explain this bias. Relativized Minimality and Dependency Locality Theory have received the most attention (Zhang, 2015; Hu et al., 2016). The former focuses on the distance between components in linear processing. The latter, which is based on the underlying structure, believes that movement across a greater number of components necessitates a higher level of cognitive consumption (Rizzi, 1990; Hu et al., 2016).

There is no agreed conclusion in the studies on Chinese relative clauses: some studies have found the advantage of subject clauses (Liu et al., 2011; Wei and Chen, 2020). Some studies prove that object clauses have advantages in acquisition and processing (Feng and Wang, 2013; Wang and Bing, 2013). These studies not only examine the processing of relative clauses in native Chinese speakers but also examine the development and processing of relative clauses in second and third language learners, as well as individuals with language impairments (He and Yu, 2001). Wei and Chen (2020) investigated the acquisition of relative clauses by high-level English learners in China through a Chinese-English translation test. Their study found out that the scores of Chinese English learners in translating subject relative clauses are significantly higher than that of object relative clauses. The main effect of clause types is significant, indicating that the two kinds of relative clauses are different in acquisition and actual use. Their research also examined the influence of position (clauses occupy subject or object position) on the acquisition and found out that subject-position relative clauses are easier to be learned.

As the Chinese relative clause has a specific word order, it is an excellent syntactic structure to test the word order hypothesis in the Matrix Language Framework (Myers-Scotton, 1993), which proposes that the word order of the embedded language should be consistent with that of the matrix language. At the same time, with different complexity of subject and object clauses, our research will use it to test the influence of grammatical difficulties on code-switching, which is also a test for Dependency Locality Theory and learners’ acquisition of relative clauses.

### 1.3. Code-switching on relative clauses

According to the principle of morpheme order in MLF, the word order of the embedded language should be consistent with that of the matrix language. Therefore, when code-switching is in relative clauses, the word order of the embedded language should be the same as the matrix language. Chinese and English relative clauses build up the word orders with opposite movement directions. By investigating code-switching occurring in the movement, this study aims to observe whether a cost is incurred during syntactic processing or not. As Chinese relative clause (clause-antecedent) is different from the English word order (antecedent-clause), this study chooses the opposite word orders of the relative clause to investigate Chinese-English code-switching costs in syntactic processing. The word order of Chinese relative clauses is unique in typology. Among the 879 languages in Dryer (2013), there are only five languages with VO and Rel-N, including Chinese, as shown in example (2a). Meanwhile VO and N-Rel co-occurs in 416 languages, including English in example (2b).

1. a.*wo renshi dai maozi de shushu*.
  I know wear a hat aux uncle
  b.I know this uncle who wears a hat.

Examples (2a) and (2b) mean the same. The word order of the Chinese relative clause in the sentence a is “verb + noun + *de* + antecedent,” where *de* is the marker of the Chinese relative clause, which is in front of the antecedent. In the sentence b, the word order is “antecedent +who+ verb + noun,” where “who” is the marker of the English relative clause, which is placed after the antecedent.

According to the principle of morpheme order, when Chinese and English code-switching occurs in the relative clause with only one embedded word, the sentence order should be consistent with the matrix language. Therefore, if the matrix language is English with an embedded Chinese relative clause, the word order of the Chinese clause should be the same as in English, with the clause coming after the antecedent. As is shown in example (3).

1. a.I knew this uncle who wore *maozi* (hat).
  b. *I knew this wore *maozi* (hat) *de* (aux) uncle.

**Figure 1:**
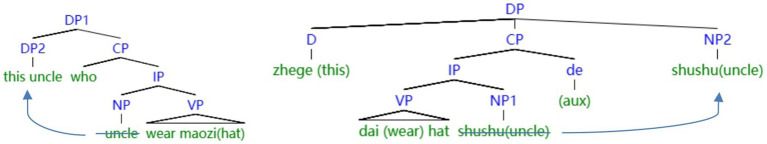
Tree structures of CS relative clauses.

Example (3a) and (3b) are two sentences that includes English-Chinese code-switching, where English is the matrix language, and Chinese is the embedded language. The correct word order should be the one in English (3a). Whereas sentence (3b) has a Chinese noun in the relative clause with the Chinese word order, and is not in conformity with the matrix language which may produce cost in processing. The difference between the two sentences (3a and b) is only in the direction of the antecedent movement. The direction of sentence (3a) is the same as that of the English relative clause, which has moved from the base generation position (in front of wear) to the left before WHO, while the direction of the sentence (3b) is the same as that of the Chinese relative clause (Myers-Scotton and Jake, 2009). At the same time, the movement distances in both CS sentences (3a and 3b) are the same, and there is no need to cross other components (see Figure 1). Therefore, when the direction of movement affects the code-switching costs, it suggests that the cost may come from the sentence processing stage.

### 1.4. The present study

Previous research has examined the lexical-level costs of code-switching. Some theories and empirical research also provide evidence that syntactic processing may be a source of costs associated with code-switching. However, very few, if any, studies have directly looked at the costs at the syntactic level. The current study thus aims to examine whether costs are from switching in syntactic processing.

The current study conducts two experiments with self-paced reading and acceptability judgment tasks to see if the correspondence of the matrix language to movement directions affects code-switching costs. The results indicate that costs may or may not be elicited during syntactic processing.

Several factors, such as domestic languages, matrix languages, and tasks, have an impact on the costs (Chang et al., 2017). Therefore, Chinese-English and English-Chinese bilinguals, as well as code-switching from two directions (English to Chinese or the other way round), are all included in this study. Additionally, it employs offline and online tasks. We predict that costs will be present in a number of situations, demonstrating syntactic processing as a source of code-switching costs.

## 2. Experiment 1: code-switching on the relative clauses in the object position

### 2.1. Design

Experiment 1 is a 2 × 2 two-factor repeated measurement design. The first independent variable is word order, a within-subject design with two levels: Chinese and English order. The second independent variable is the matrix languages, a within-subject design with two levels: Chinese and English. The dependent variable is the acceptability in Acceptability judgment task (shortened as AJT) and RT in self-paced reading.

### 2.2. Participants

Twenty-six Chinese-English (Chinese native English learners, shortened as Ch-En) bilinguals participated in self-paced reading and acceptability judgment tasks. They are mainly undergraduates in Beijing with an age range of 18–30. The English proficiencies of participants are upper intermediate (self-rated score 6.26/10). The Wilcoxon signed rank test showed a significant difference between the Chinese-English self-assessment, *p* < 0.001.

After the experiment, each participant received 30 *yuan*, and an additional 15 *yuan* as a reward when the accuracy was over 95%. The study was approved by the School of Chinese as a Second Language, Peking University.

### 2.3. Materials

Thirty-two Chinese sentences containing object-position relative clauses, as well as their English translations, are the original materials in the study, seen in Supplementary material. Some expressions are modified in the non-interest region of English sentences. The acceptability of sentences and the word frequencies of corresponding Chinese and English components in relative clauses are controlled.

This study only switches one noun in each relative clause and does not constitute any island to satisfy the Morpheme Order Principle. Additionally, to make subjects activate both languages before the relative clause, the determiner in the antecedent DP is switched. And to prevent the participants from guessing the purpose, parts of the following sentence are also switched. Finally, the word orders of both Chinese as matrix language, English as embedded one (Ch-En) and En-Ch relative clauses are switched to the embedded-language word orders or kept as the original ones to make up all four conditions in the experiment. Each group of sentences has one comprehension question, and the language of comprehension questions is consistent with the matrix language of the experimental sentences. Table 1 is the examples, and all Chinese words are Chinese characters (but Pinyin here for easy access).

**Table 1:** Examples of materials in Ex1.

Types	Sentences
En-Ch En order	I know *这个* **gentleman who always wears** *眼镜*. He is a *朋友*of my father’s.
	这个-zhege-this; 眼镜-yanjing-glasses; 朋友-pengyou-friend.
En-Ch Ch order	*I know *这个* **always wears** *眼镜/的* **gentleman**. He is a *朋友* of my father’s.
	的-de-AUX.
Ch-En Ch order	*我/认识*/**this/** *总是/戴* **/glasses/** *的/叔叔*, */他/是/我/爸爸的*/friend.
	我-wo-I; 认识-renshi-know; 总是-zongshi-always; 戴-dai-wear; 叔叔-shushu-gentleman; 他-ta-he; 是-shi-is; 爸爸-baba-father.
Ch-En En order	*我/认识/**this/** *叔叔* **/who/** *总是/戴/* **glasses**, */他/是/我/爸爸的*/friend.
Comprehension Q	My father has a friend.—T

The underlined and bold parts are the areas of interest. The italic parts are Chinese words, and Pinyin (pronunciation) and translation are under the sentences. The slash lines show the Chinese word segmentation. The sentences with * are theoretically incorrect sentences.

Experimental materials form four groups, with 32 sentences in each group and eight in each type. Each participant could only see one sentence in a group. At the same time, participants also read 64 filler sentences with comprehension questions. 96 sentences are presented pseudo-randomly to ensure that three experimental sentences do not appear in a row.

### 2.4. Procedure

Before the experiment, the participants completed the Chinese and English proficiency tests and get familiar with the word list of the experimental sentences.

**Figure 2:**
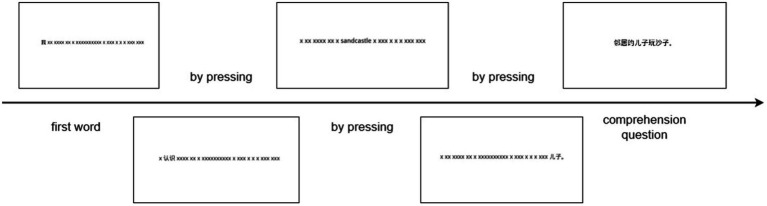
The illustration of the experiments.

OpenSesame V3.3.5 is used to present the program and record the data for the self-paced reading. The instruction is presented first, and then the practice session. The experimental sentence is presented in the non-cumulative form of a moving window. The first word is presented while other words are presented as “x” (each English word is replaced by an x. Each Chinese character uses two x instead), and then the word is presented by pressing the space bar. At the same time, the previous one disappears. After the whole sentence is presented, the reading comprehension question will appear on the next screen after a press. The participants press “Z” or “/” to represent “yes” or “no” to answer the questions. The illustration is in Figure 2.

After the online experiment, the participants complete the acceptability judgment of the experimental sentences and fillers (seven-point scale). Each participant judges 96 new sentences.

### 2.5. Data analysis

The correct rate of reading comprehension tasks is 95.99% (*SD* = 2.10%). For the reaction time, we first delete incorrect reactions and remove outliers.

SPSS 20.0 and R 3.6.3 are used for data statistics: SPSS 20.0 is used for descriptive statistical analysis, paired *t*-test, and repeated measurement test. The POLR function in the MASS package in R is used to conduct logistics analysis on the acceptability judgment data. And the LME4 package (Bates et al., 2015) does linear mixed-effect model analysis on the experimental data. In the mixed effects model analysis by R, logarithms reaction time is used.

### 2.6. Results

#### 2.6.1. Acceptability judgment task

The descriptive statistical results are shown in Table 2. Repeated measurement variance analysis by the subject shows that the main effect of the matrix language is not significant, and the main effect of consistency is near significant [*F_1_*(1, 25) = 4.123, *p* = 0.053, *η^2^* = 0.142, *β* = 0.497]. The interaction is significant, *F_1_*(1, 25) = 4.306, *p* = 0.048, *η^2^* = 0.147, *β* = 0.514. The results of simple effect analysis show that when Chinese is the matrix language, there are significant differences between the consistent and inconsistent groups [*p* = 0.005, *CI* [0.078, 0.384], *η^2^* = 0.279, *β* = 0.848]. When English is the matrix language, there is no significant difference in main effects and interaction effects. Repeated measurement variance analysis by item also finds a near significant main effect of consistency. The results show that consistency influences native speakers’ acceptability of code-switching sentences.

**Table 2:** descriptive statistics results of Ex 1.

ML	Agree	Disagree
Chinese	3.05(1.46)	2.82(1.41)
English	2.80(1.43)	2.75(1.42)

#### 2.6.2. Self-paced reading

Descriptive statistics results from each region of interest are shown in Table 3. The results of linear mixed model analysis are in Supplementary material.

**Table 3:** Descriptive results of reaction time in Ex 1 (ms, and SD in brackets).

ROI	Ch-En agree	Ch-En disagree	En-Ch agree	En-Ch disagree
D	388.87(161.20)	376.79(148.80)	400.94(122.41)	403.65(118.85)
V	414.42(153.22)	389.11(127.43)	521.77(198.14)	513.96(227.01)
Inner-n	573.71(325.71)	574.84(362.58)	610.40(319.16)	541.67(240.00)
Outer-n	453.58(191.89)	425.72(155.72)	494.52(252.85)	513.98(243.82)

In the antecedent D (*zhege*/this) region, the linear mixed model analysis shows that the main effect of matrix language is significant, the main effect of consistency and the interaction are not significant.

In order to examine the individual effects of the matrix and native languages, a simple effect analysis is performed, even in cases where the interaction is not found to be significant. The results of the simple effect analysis show no significant difference between the consistent and inconsistent groups when Chinese and English are the matrix language, respectively.

In the verb (*dai*/wears) region, the linear mixed model analysis shows that the main effect of subject language is significant, the main effect of consistency is not significant, and the interaction between the two is not significant. The results of the simple effect analysis show no significant difference between the consistent and inconsistent groups when Chinese and English are the matrix language, respectively.

In the switched “inner N” (glasses/*yanjing*) region, the linear mixed model analysis shows that the main effect of matrix language is significant, the main effect of consistency is not significant, and the interaction between the two is significant. The results of simple effect analysis show that when Chinese is the matrix language, there is no significant difference between the consistent and inconsistent groups. When English is the matrix language, there is a significant difference between the consistent and inconsistent groups [*t*(662) = 2.837, *p* = 0.005].

In the “outer N” (*shushu*/gentleman) region, the results of linear mixed model analysis show that the main effect of matrix language is significant, the main effect of consistency is near significant, and the interaction between the two is significant. The results of simple effect analysis show that when Chinese is the matrix language, there is a nearly significant difference between the consistent and inconsistent groups [*t*(636) = 1.710, *p* = 0.088]. When English is the matrix language, there is no significant difference between the consistent and inconsistent groups.

### 2.7. Discussion

The findings demonstrate that the code-switching cost is significantly influenced by whether the relative clause word order is consistent with the matrix language. AJT results show that when the relative clause word order is consistent with the matrix language, bilinguals are more likely to accept the switching sentences. In other case, when the matrix language is Chinese, Ch-En bilinguals are more sensitive to it. Self-paced reading provides more details on time course: the main effect of consistency and the interaction between consistency and matrix languages appear in the outer N period. The interaction also appears in the switched inner N area. Last but not least, Ch-En bilinguals performed differently when the matrix languages are different.

The cost of inconsistency, found in both the acceptable judgment task and the self-paced reading task, indicates that the word order of the relative clause affects CS cost. From this, we can infer that the cost comes from the word-order processing of relative clauses at the sentence-processing stage. Chinese and English declarative sentences have the same SVO word order, but the orders of the relative clause are not consistent. The movement directions of the Chinese and English relative clauses are not the same. During the movement, Chinese goes to the right while English to the left, which is the main difference between Chinese and English relative clauses (Xiong, 2005). In Experiment 1, code-switching costs occur in the Noun-movement where the opposite moving need to be processed. The results also show that the Noun-movement is one of the code-switching cost sources.

In Experiment 1, some results do not follow the expectations: in the “inner N” region, when English is the matrix language, and in the “outer N” and “V” region, when Chinese is the matrix language, the inconsistent condition processes faster. These phenomena may be due to the unclear definition of the matrix languages or the sentence ending effect.

First, there are many possibilities for the matrix languages of relative clauses. The definition of the matrix language of a clause is controversial, which may be the language of the main clause or the language used by the core component of the clause. At present, the research on code-switching has not provided a certain confirmation method to define the matrix language of subordinate clauses (Myers-Scotton, 1993). In this study, the content words in the core are consistent with the matrix language, and thus we define the language of the main clause as the matrix language of the relative clause. For example, in “*Wo renshi* (I know) this *zongshi* (always) *dai* (wear) glasses *de* (aux) *shushu* (gentleman), *ta shi wobaba de* (he is my father’s) friend”(Chinese in italics, and translation in brackets), the language of the main clause “*wo renshi* (I know)” and the content word “*shushu* (gentleman)” are Chinese, so the matrix language is Chinese, and the word order of the clause should follow the Chinese.

However, there are other possibilities for the matrix language of relative clauses. One is the language of the antecedent’s determiner. The core component of the relative clause is DP, that is, “this” in the sentences. To enable the participants to activate both languages before entering the relative clause, “this (*zhege*)” is switched in this study, which may lead to the change of the matrix language, resulting in costs contrary to the prediction. This possibility could explain some of the non-conformity, but not all. Experiment 2 clarifies the matrix language by changing the switching position in the main sentence.

Another possibility for the matrix language is the mother tongue of the bilinguals. Previous studies have proven that proficiencies affect cognitive control abilities and code-switching costs (Meuter and Allport, 1999; Costa and Santesteban, 2004; Zhang and Cui, 2008; Linck et al., 2012). Therefore, Chinese-English bilinguals have higher rate of activation of their mother tongue with the dominant word order from Chinese. The inconsistency condition (Chinese order) processes faster when English is the matrix language. This possibility is also limited as it cannot explain the processing advantage of the inconsistency condition when Chinese is the dominant language. To solve this problem, Experiment 2 includes both English and Chinese bilinguals.

The phenomenon may also relate to the sentence-ending effect. In this experiment, the relative clause is at the end of the first clause. Although the participants know the sentence does not end after the relative clauses, they are still likely to have a long pause when reading punctuation marks, showing the sentence-ending effect (Ni et al., 1998; Chang et al., 2009). Moreover, by the end of the sentence, bilinguals, acquiring enough information, may start to integrate information causing the reactions to delay. The sentence ending effect can explain parts of inconsistent advantages. However, it cannot explain the inconsistent advantage in the V region or the performance of the “outer N” when Chinese is the matrix language and “inner N” when English is the matrix. In addition, previous studies have found that punctuation processing tends to trigger skip reading and shortened fixation time (Wang et al., 2018). This facilitating effect is inconsistent with the sentence ending effect found in this study. In view of this possibility, the positions of relative clauses change in Experiment 2, to eliminate the possible influence of punctuation or sentence-ending effect.

## 3. Experiment 2: code-switching on the relative clauses in the subject position

To further examine the costs from syntactic processing, we design experiment 2 for Chinese-English and English-Chinese (shortened as En-Ch) bilinguals to eliminate the effects of matrix languages and sentence structures with updated materials.

### 3.1. Design

This experiment is a 2 × 2 × 2 three-factor repeated measurement design. The first independent variable is word order, a within-subject design with two levels: Chinese and English. The second independent variable is the matrix language, a within-subject design with two levels: Chinese and English too. The third variable is the language background, a between-subject design with two levels: Chinese-English and English-Chinese bilinguals. The dependent variable is acceptability in AJT and RT in self-paced reading.

### 3.2. Participants

Twenty-one Chinese-English and 16 English-Chinese bilinguals (shortened as En-Ch) participated in self-paced reading and acceptability judgment tasks. Ch-En bilinguals are students in Beijing and they participated in the experiment in the lab. En-Ch bilinguals were recruited online with an age range of 18–35. They conducted the experiments remotely under experimenter’s supervision by Tencent Meeting.

These bilinguals are upper-intermediate learners of Chinese or English. The differences within the in-group regarding bilingual proficiencies are significant. After the experiment, each participant receives 30 *yuan*, and an additional 15 *yuan* as a reward when the accuracy is over 95%.

### 3.3. Materials

We revised the sentences in Experiment 1. First, the main clauses are extended to “I heard from XX (name) that.” Second, the relative clauses move to the subject position of the indirect speech, e.g., “I heard from my father that the uncle always wears glasses ….” Finally, the predicate is added directly without punctuations, such as “plans to come to my house and have dinner next week “. The whole sentence is “I heard from my father that *the gentleman who always wears glasses* plans to come to my house and have dinner next week” (The italics are relative clauses and their antecedents, including the four areas of interest in this experiment). All sentences without switching are in Supplementary material.

Thirty-two Chinese sentences containing object-position relative clauses are the original materials in the study, as well as their English translations. Then we make switching materials with the same steps in Experiment 1. Table 4 is the examples for the materials in Experiment 2.

**Table 4:** Examples of materials in Ex 2.

Types	Sentences
En-Ch En order	I heard from *爸爸* that **the gentleman who always wears** *眼镜* plans to come to my house *吃饭* next week.
	爸爸-baba-father; 眼镜-yanjing-glasses; 吃饭-chifan-have dinner.
En-Ch Ch order	*I heard from *爸爸* that **this always wears** *眼镜/的* **gentleman** plans to come to my house *吃饭* next week.
	的-de-AUX.
Ch-En Ch order	*我/听*/my father/说*/**那个/总是/戴/* **glasses** */的/叔叔**/下周/要/来我家, /和/我们/一起/*have dinner。
	我-wo-I; 听-ting-hear from; 说-shuo-say; 那个-nage-that; 总是-zongshi-always; 戴-dai-wear; 叔叔-shushu-gentleman; 下周-xiazhou-next week; 要-yao-will; 来我家-lai wojia-come to my home; 和-he-with; 我们-women-we; 一起-yiqi-together.
Ch-En En order	*我/听/my father/*说**/那个/叔叔* **/who/** *总是/戴* **/glasses**/*下周/要/来我家，/和/我们/一起*/have dinner。
Comprehension Q	A gentleman is coming to my house next week. —T

The underlined and bold part is the area of interest. The italic parts are Chinese words, and Pinyin (pronunciation) and translation are under the sentences. The slash lines show the Chinese word segmentation. The sentences with * are theoretically incorrect sentences.

### 3.4. Procedure and data analysis

The procedure and data analysis are the same as in Experiment 1. The average correct rate of reading comprehension tasks is 95.54% (*SD* = 2.10%).

### 3.5. Results

#### 3.5.1. Acceptability judgment task

The descriptive statistical results are in Table 5. For Ch-En bilinguals, the logistic regression model for ordered variables analysis shows that the main effect of the matrix language is not significant; The main effect of consistency is significant (*β* = 1.503, *t* = 7.562, *p* < 0.001, OR value 4.494). The interaction is significant (*β* = −1.021, *t* = 7.562, *p* < 0.001, OR value 4.494). The results of simple effect analysis show that when Chinese is the matrix language, there are significant differences between the consistent and inconsistent groups (*Z* = 7.562, *p* < 0.001). When English is the matrix language, there are also significant differences between the consistent and inconsistent groups (*Z* = 2.441, *p* = 0.015). Ch-En bilinguals are sensitive to the relative clauses’ word orders.

**Table 5:** Descriptive statistics results of Ex 2 (ms, *SD* in blankets).

	ML	Agree	Disagree
Ch-En bilingual	Chinese	3.80(1.24)	2.64(1.28)
	English	3.13(1.47)	2.76(1.32)
En-Ch	Chinese	3.87(1.36)	3.75(1.45)
Bilingual	English	3.85(1.36)	3.86(1.40)

For En-Ch bilinguals, the logistic regression model for ordered variables analysis finds no significant effect of the matrix language, consistency, or interaction. En-Ch bilinguals exhibit a lack of responsiveness to the relative clause word order’s conformity to the matrix language.

#### 3.5.2. Self-paced reading

Descriptive statistics results of each region of interest are shown in Table 6. The results of linear mixed model analysis are in Supplementary material.

**Table 6:** Descriptive results of reaction time in Ex 2.

	ROI	Ch-En agree	Ch-En disagree	En-Ch agree	En-Ch disagree
Ch-En bilingual	D	396.66(135.29)	391.63(156.23)	398.72(156.26)	419.88(158.72)
	V	394.19(157.82)	414.60(164.54)	475.45(192.52)	502.61(214.96)
	Inner-n	608.56(358.86)	609.75(360.16)	577.96(290.40)	639.75(327.99)
	Outer-n	443.86(210.01)	406.36(204.10)	530.09(250.31)	561.84(274.43)
En-Ch bilingual	D	371.65(140.78)	374.72(151.89)	343.05(138.05)	335.81(124.44)
	V	359.04(149.16)	349.29(136.70)	353.90(134.39)	343.45(127.56)
	Inner-n	435.63(211.38)	441.03(223.73)	414.22(226.04)	484.22(250.69)
	Outer-n	391.47(183.40)	399.93 (188.04)	369.58(154.51)	385.19(156.42)

In the antecedent D (*zhege*/this) region, the linear mixed model analysis shows that the interaction between matrix language and consistency is near significant. But all main effects and other interaction effects are not significant. The results indicate the validity of the experiment and analysis.

In the verb V (*dai*/wears) region, the linear mixed model analysis shows that the main effects of matrix language and the interaction between matrix languages and background are significant. The matrix language and background influence bilinguals’ code-switching processing.

In the noun “inner N” (glasses/*yanjing*) region, the linear mixed model analysis shows no significant effect. For Ch-En and En-Ch bilinguals respectively, but there are significant differences when English is the matrix language.

In the noun “outer N” (*shushu*/gentleman) region, the results of linear mixed model analysis show that the main effect of consistency is nearly significant, and the interaction between the matrix languages and consistency is significant. For Ch-En bilinguals, the main effect of matrix language, consistency, and their interaction are significant. When Chinese is the matrix language, the effect of consistency is significant. But for En-Ch bilinguals, only the effect of matrix language is found.

For the whole relative clauses region, significance is found in the main effect of the matrix languages and the interaction between the matrix languages and background. For En-Ch bilinguals, the interaction between consistency and matrix language is significant, and the consistency effect is significant when English is the matrix language.

Bootstrap paired sample *t*-tests are conducted with a sample size of 10,000 to analyze the data of Chinese-English bilinguals under the condition of consistency and inconsistency. The results show that in the “V” and “outer N” regions, the consistency effect in Chinese is nearly significant by subject analysis. In the “inner N” region, the consistency effect is significant when English is the matrix language.

### 3.6. Discussion

Experiment 2 improves the materials and resolves remaining problems in Experiment 1. Overall, the findings are in line with the theoretical hypothesis.

Some conjectures from Ex1 are verified. First, Experiment 2 provides a clear definition of matrix language. The main clause’s objects are the first portions of the sentences to switch, which are independent from the relative clause and have no bearing on the relative clause’s choice of matrix language. Experiment 2 increases the number of components additionally in the section prior to the RC from 3 to 5, which makes the matrix language exactly the one used in the main clause. The findings demonstrate that when English is the matrix language, condition with no inconsistency has an advantage. Additionally, the matrix language, not the linguistic background, has a large impact on the outer N and V areas. With the results of Experiment 1, we conclude that the matrix language of a relative clause may be determined by the language used by the core component and antecedent DP (the/this/that), not the mother tongues of bilinguals.

Second, Experiment 2 excludes the sentence ending effect caused by punctuation. In Experiment 2, the relative clause serves as the indirect speech’s subject and is followed by a predicate component without punctuation. The results of Experiment 2 did not find the ending effect of pauses in the relative clause, indicating that punctuation may bring the end effect. This result goes against the earlier findings (Wang et al., 2018). However, due to the lack of other relevant evidence on punctuation processing, the role of punctuation in language processing needs to be further investigated.

The modified experiment illuminates the role of consistency. The consistency effect in AJT is significant. In self-paced reading, the Chinese-English and English-Chinese bilinguals exhibit significant consistency effects in some areas. It demonstrates how the matrix language and word order have an impact on code-switching costs. In this study, the word order of relative clauses—basically the direction of movement—is manipulated. Nouns should be relativized to the right when Chinese is the matrix language and to the left when English is the matrix language. Movement is an operation in the syntax formulator. We can conclude that code-switching in the syntax formulator results in costs as we discover the cost of movement processing.

Experiment 2 finds the code-switching costs have different patterns when matrix languages, language proficiencies and tasks are diversified. When English is the matrix language, Ch-En and En-Ch process more quickly in the inner N (glasses) region. But when Chinese is the matrix language, there are no effects. When processing relative clauses including code-switching, bilinguals need to process code-switching and relationalization together, which requires high consumption. The inner N is the last word in relative clauses when English is the matrix language. Bilinguals can quickly process relative clauses after processing this word. They are intelligent enough to notice the contradiction as a result. However, when Chinese is the matrix language, inner N is in the middle. Bilinguals need to get more input to conduct movement and processing RC. Therefore, they are insensitive to the inconsistency. The proficiency of matrix languages is also important. Bilinguals have different control capacities over two languages during complex processing (Schilling et al., 2014). The fact that bilinguals who are En-Ch have consistency effect when English is the matrix language is the clue.

The offline AJT shows significant effects, especially when Chinese is the matrix language. In the online self-paced reading task, the consistency effect sizes are small. The results are different from Ex1. These phenomena may be due to task and structure difficulties. In experiment 2, we moved the relative clause from the object position to the subject. Previous studies have shown that for children and second language speakers, the subject relative clause in the subject position is the easiest (Ma, 2012; Feng and Wang, 2013). Structural difficulty reduction helps bilinguals to process inconsistencies in online tasks, while they could find inconsistencies easily in offline tasks. The structural difficulty affects the generation of code-switching costs (Tarłowski et al., 2013; Zeller, 2020). If bilinguals have enough cognition resources to analyze the syntactic structure in AJT, they can fully invoke explicit knowledge to make judgments and are more sensitive to consistency differences. Especially when the matrix language is the mother tongue of bilinguals, they make decisions quicker and more accurate.

There is a finding that our prediction cannot explain: When Chinese is the matrix language, Ch-En’s RT to the consistent condition is slower in “outer N (uncle).” This phenomenon may be related to the unique relative clauses to Chinese. In most languages, with SVO as the basic word order and noun-relative clause order, the core components are in the front of the RC. However, Chinese is an SVO language with a postpositional antecedent, with core components on different positions between the main sentence and relative clauses. Chinese native speakers can accept two word-orders. When Chinese and English are activated, Ch-En bilinguals activate Chinese and English basic word order with a prepositive core component, English and Chinese RC order. In real-time language processing system, the order of the prepositive core component may have an advantage. It may be why they can better understand the relative clause with the prepositive core component. However, this supposition lacks evidence. The reason why Ch-En bilinguals have fast processing in the condition of inconsistent “outer N (uncle)” position needs to be investigated.

## 4. General discussion

Experiments 1 and 2 investigate the Chinese-English code-switching costs in syntax processing by manipulating the relative clause word orders. The results show that costs are from the processing of movement, which indicates the syntax process is one of the sources of code-switching costs. Also, the experiments also show evidence of RC’s processing mechanism.

### 4.1. The source of code-switching costs in syntax processing

This study uses two experiments to examine the code-switching costs in syntax processing. The results show that when relative clause word order is inconsistent with the matrix language, code-switching produces more costs with slower processing. As the order processing is at the stage of the syntactic operation, this stage is the source of code-switching costs. This result conforms to the prediction of the Morpheme-Order Principle in the Matrix Language Framework, Uniform Structure Principle, and Differential Access Hypothesis (Myers-Scotton and Jake, 2009).

According to the Uniform Structure Principle, code-switching should keep in consistency with the matrix language generally. The Different Access Hypothesis holds that different morphemes have different switching mechanisms: The bridging late system morphemes, external late system morphemes, and other syntax structures are prominent in the syntactic generator. Morphemes salient in different portions have different difficulty degrees in code-switching: the morphemes generated in the mental lexicon can switch automatically, while the morphemes and other operators in the syntactic generation cannot switch, and understanding these switching structures is difficult with extra cognition resources. Therefore, code-switching in the process of movement is unnatural and labeled, requiring additional cognitive resources and resulting in consumption. That is to say, the code-switching cost will occur on the processing of switched syntax structures. The results of these experiments confirm the hypothesis and show that the syntax process is one of the sources of code-switching costs, which is in line with the predictions of the Matrix Language Framework Model (Myers-Scotton, 1993).

The component realized in syntactic generators is less code-switched. According to definition of external late system morphemes of Myers-Scotton (2002), there are no such morphemes in Chinese or English. Given that the one function of late system morphemes is to construct sentences, this study aims to examine the associated costs by selecting the word order of the relative clause as a means of testing the effects of switching which involves movement in sentence completion. The Morpheme Order Principle also suggests that the word order of the embedded language should be consistent with that of the matrix language. The result from this study, the switching on relative clauses producing cost, shows that the sentence completing stage is the source of cost. This result conforms to the prediction of the Morpheme Order Principle in Matrix Language Framework as well as the Unified Structure Principle and Different Access Hypotheses (Myers-Scotton and Jake, 2009) and reflects the process of sentence-level code-switching.

However, the results in these two experiments are not consistent, indicating some factors influencing the production of costs. Matrix languages and the difficulty of the task could be two major determining factors. First, CS costs are influenced by the matrix language’s proficiency. When the matrix languages change, bilinguals between Chinese and English show varying effect sizes in various regions. Bilinguals have different control abilities when processing languages because their language proficiency is different (Schilling et al., 2014). Participants in this study process language, code-switching, and movement together. Native speakers of matrix languages are better at processing language and movement. That is why Chinese native speakers exhibit a sensitive perception of consistency differences when Chinese is the matrix language. Also, the costs are impacted by the complexity of the task. When the structure is simple (Ex1), bilinguals have enough cognitive resources to process the switching quickly online. However, bilinguals hardly understand sentences automatically when dealing with a more complicated relative clause nested inside the indirect speech clause (Ex2). Therefore, they may ignore the inconsistency during online processing. When they have enough time to think about linguistic structure and the built-in grammar of code-switching, they notice inconsistency and make better decisions offline.

### 4.2. The mechanism of relative clauses processing

Researchers have proposed different theoretical models to explain the generation of relative clauses, among which the Relativized Minimality Theory and the Dependency Locality Theory are the two most widely discussed (Zhang, 2015; Hu et al., 2016). Many empirical studies on relative clauses provide evidence for these two theories. This study provides evidence from Chinese-English bilinguals and supports Dependency Locality Theory.

**Figure 3:**
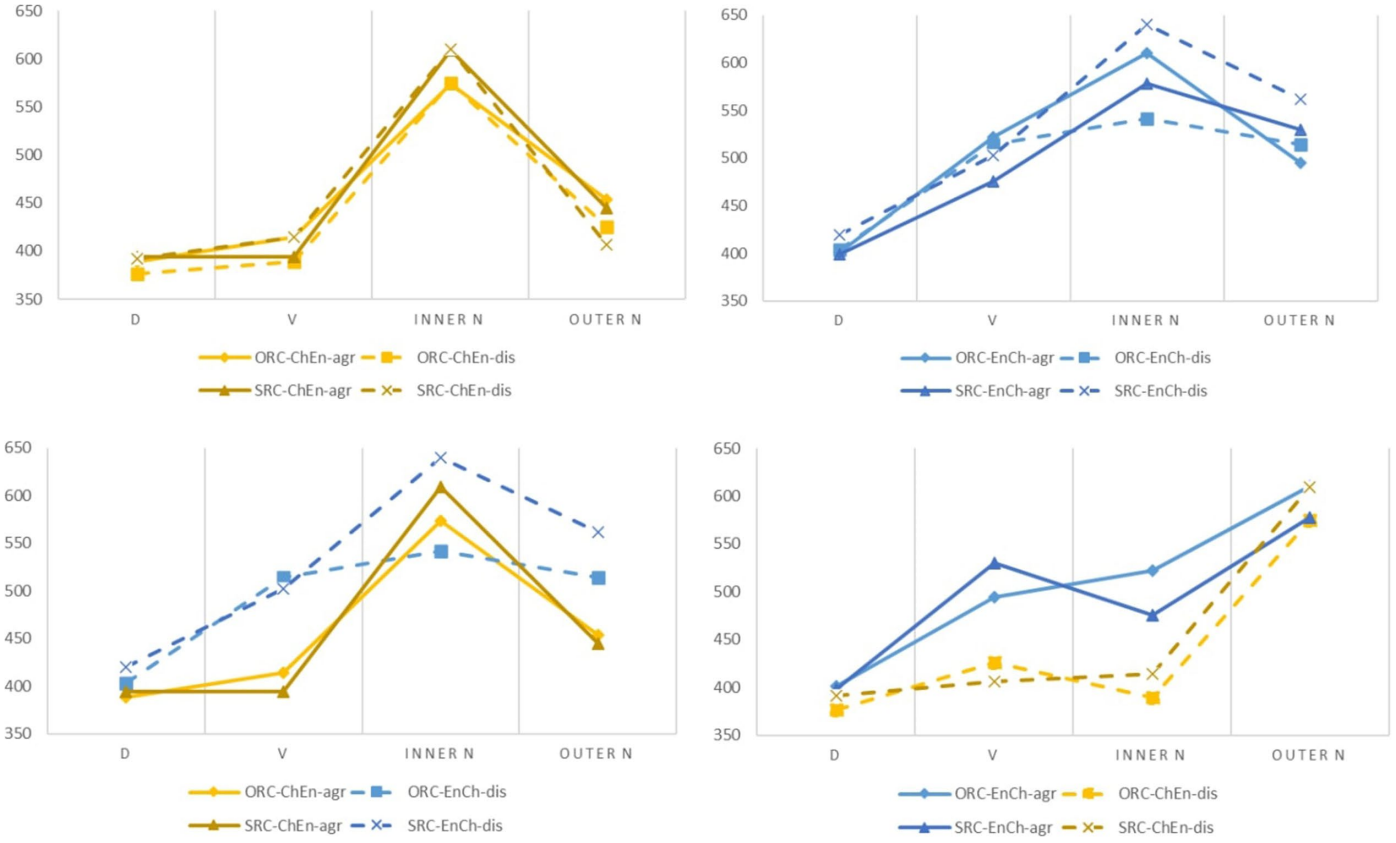
Processing diagram of relative clause (The two graphs above are in the order of the underlying structure. The two graphs below are in the order of time course).

The word orders of relative clauses are manipulated in two experiments. In the sentences, the words “always” “wear” “*yanjing* (glasses)” and “gentleman” are used to make relative clauses “gentleman who always wears *yanjing* (glasses)” and “always wears *yanjing* (glasses) *de* (aux) gentleman” respectively in the Chinese or English relative clause word order. During statistics, we draw the processing diagram according to the underlying structure order and the time course, respectively (see Figure 3).

Based on the underlying structure (the figures above), the response patterns of relative clauses are relatively consistent. Although the sentences from the two conditions are not the same in the linear with different movement directions, the corresponding components have similar processing trends. However, based on the surface structures order (the figures below), the reaction patterns of relative clauses are generally different. It can be inferred that the time course may not be the most important point for the processing. The overall trend is that the processing of switched inner N (glasses) becomes slower, reflecting the code-switching cost. At the same time, the patterns may also be related to the matrix language. We also control the frequency of words corresponding to the same position, which eliminated the influence of vocabulary and language differences to a certain extent.

According to the patterns in the Experiments, we find out that the process of the relative clause may be more related to the underlying structure rather than to the surface time course. Though the experiments use self-paced reading in which the participants read word by word according to the time course, the results show a more consistent underlying reading pattern, indicating that when understanding relative clauses, bilinguals tend to use the underlying structure. Reading comprehension starts from the underlying grammatical structure (Peng, 1991). It also indicates that noun movements are in the processing of relative clauses. This study supports that the underlying structure plays a role in relative clause processing, consistent with the predictions of the Dependency Locality Theory (Rizzi, 1990). These experiments also prove the rationales for the direct comparisons of corresponding components (Inner-n, Outer-n, V) during data analysis.

## 5. Conclusion

This study uses relative clause order as a maker to look at the code-switching cost in syntactic processing. The findings imply that matrix language should be consistent with the relative clause order. If not, the understanding of code-switching produces cost. The syntactic operation, which includes word order processing and movement, is one of the sources of code-switching costs. Our finding is consistent with the predictions of the Morpheme-Order Principle in the Matrix Language Framework, the Uniform Structure Principle, and the Differential Access Hypothesis (Myers-Scotton and Jake, 2009).

Experiment 2 is a modification of Experiment 1. Comparing the two, we believe that the matrix language is mainly decided by the core component of the clause, rather than the learner’s mother tongue. In addition, Experiment 2 also eliminated the sentence end effect. Previous studies on sentence end effects often focus on the end of the sentences, rather than the punctuation in the middle. The results of this experiment also suggest that researchers need to pay more attention to the processing of punctuation.

The processing patterns in the Experiments show that Chinese and English bilinguals rely on the underlying structure when understanding relative clauses. Even when reading relative clauses word by word, bilinguals tended to process the relative clauses from the bottom-up pattern. The finding of this pattern provides bilingual evidence for the Dependency Locality Theory. Furthermore, the experiments also indicate that the subjects’ language proficiency, as well as the complexity of the structures and tasks involved, may affect the understanding of code-switching.

This study investigates code-switching on the syntactic level, which gives new evidence on the source of code-switching costs. At the same time, this study provides new clues for several theoretical questions, such as the definition of matrix language in clauses, and the mechanism of relative clause processing from a bilingual perspective. Finally, this study finds several linguistic factors may have influence on code-switching costs. Some of them are with less concern before.

This work presented several limitations. First, there are not enough participants, particularly En-Cn bilinguals. The majority of them carried out the experiments online, where there may have been more disturbance than in the lab. Second, because only Chinese and English were used to test the source in this study, the findings need to be verified in more language conditions. Therefore, further investigation is needed to examine the conclusion with more languages and grammatical structures.

In conclusion, this study investigates code-switching at the syntactic level and provides fresh evidence regarding the origin of code-switching costs. In addition, this study offers new insights into some theoretical issues, including how relative clauses are processed from a bilingual perspective and the definition of matrix language in clauses. Finally, this study discovers that some linguistic variables may affect code-switching costs. Some of them showed less concern before.

## Data availability statement

The original contributions presented in the study are included in the article/Supplementary material, further inquiries can be directed to the corresponding author.

## Ethics statement

The studies involving human participants were reviewed and approved by YZ, Peking University. The patients/participants provided their written informed consent to participate in this study.

## Author contributions

YZ is WH’s Ph.D. supervisor. This research is a part of WH’s doctoral dissertation. WH designed and conducted the experiments, analyzed the data, and wrote the paper. YZ offered valuable guidance and assistance and revised every manuscript. WH and YZ discussed and finished the research together. All authors contributed to the article and approved the submitted version.

## Funding

This study was funded by China’s National Social Sciences Project “Application of Chinese Proficiency Grading Standards and Strategies of Textbook Research and Development” (21STA032).

## Conflict of interest

The authors declare that the research was conducted in the absence of any commercial or financial relationships that could be construed as a potential conflict of interest.

## Publisher’s note

All claims expressed in this article are solely those of the authors and do not necessarily represent those of their affiliated organizations, or those of the publisher, the editors and the reviewers. Any product that may be evaluated in this article, or claim that may be made by its manufacturer, is not guaranteed or endorsed by the publisher.
