# Supplementary material for: Code-switching costs from Chinese-English relative clauses processing
Source: Front Psychol. 2023 May 25;14:1144530. doi: 10.3389/fpsyg.2023.1144530 (PMC10249605; doi:10.3389/fpsyg.2023.1144530)
Supplement: Supplementary file 1 [file Data_Sheet_1.pdf]

# *Supplementary Material*

# Code-Switching Costs from Chinese-English Relative Clauses Processing

## 1 Supplementary materials

### 1.1 Original sentences in Ex1

1. Wo renshi zhege jingchang da wangqiu de jingli， qunian ta shi gongsi wangqiu
   I know the often plays tennis AUX manager, last year he is company’s tennis

   bisai de diyiming。
   match ‘s champion

   I know the manager who often plays tennis. He won the first prize in the company's tennis match last year.

2. Wo zhidao zhege congbu ma xiashu de laoban，tade yuangong dou feichang zunjing
   I know this never scold employee AUX boss, his employee all very respect

   ta。
   him

   I know a boss who never scolds employees. His employees hold him in high regard.

3. Wo renshi zhege congbu qing fanyi de lingdao，ta hui shuo haoduo guojiade yuyan。
   I know the never use interpreter AUX leader, she can speak many country’s language
   I know the leader who never uses interpreters. She speaks many languages.

4. Wo zhidao zhege jingchang chang minyao de nanren, ta you henduo fensi。
   I know this often sing ballad AUX man, he have many fans
   I know this man who often sings ballads. He has a big group of fans.

5. Wo renshi zhege dasuan xue fayu de xuesheng, ta xiangqu faguo liuxue。
   I know this plan to study French AUX student, she want to France study
   I know this student who will study French. She wants to study in France.

6. Wo renshi zhege jingchang ban juhui de qingnian，ta ye jingchang yaoqing wo qu tade
   I know this often hold party AUX guy, he too often invite me go to his
   juhui。
   party
   I know this often guy who often holds parties. He often invites me to his parties.

7. Wo renshi zhege zongshi bei chengyu de laowai, ta renwei zhe shi xuexi hanyu de I know this always recite idiom AUX foreigner, she think it is learn Chinese AUX good
   haofangfa。
   way.

I know this foreigner who always recites idioms. She thinks it is a good way to learn Chinese.

8. Wo renshi zhege jingchang hua ditu de daoyou, tade keren dou xihuan tade shouhui
I know this often draw map AUX guide, his guest all like his hand-drawn
ditu。
map
I know this guide who often draws maps. His hand-drawn maps are very popular with his guests.

9. Wo renshi zhege zongshi yao shouzhi de nanhai，ta jinzhang de shihou jiuhui
I know this always bite finger AUX boy, he nervous AUX time will
zheyang。
like this
I know this boy who always bites fingers. He does this when he's nervous.

10. Wo renshi zhege changchang jiao gushi de zhuanjia，ta juede gushi daibiaole zhongguo
I know this often teach poetry AUX expert, she think poety represent Chinese
wenhua。
culture
I know this expert who often teaches poetry, which she thinks represents culture.

11. Wo zhidao zhege congbu mo xiaomao de gongzhu，ta dui maomao guomin。
I know this never touch kitten AUX princess, she to cat fur allergic
I know the princess who never touches kittens. She's allergic to cat fur.

12. Wo zhidao zhege zongshi shou kuaidi de duzhe，ta meizhou douzai wangshang
I know this always receive delivery AUX reader, he every week always internet
mai zuixin chu de shu。
buy lastest publish AUX book
I know this reader who always receives deliveries. He buys the latest books on the Internet every week.

13. Wo zhidao zhege jingchang juan jiangjin de guanjun，ta meici huojiang dou huiba
I know this often donate prize AUX champion, he every time win prize all will
jiangjin juangei minjian zuzhi。
prize donate folk organization.
I know this champion who often donates prizes. He always donates all his prizes to a charity organization.

14. Wo renshi zhege dasuan chao hetong de mishu，laoban rang ta xiaban qian jiao
I know this will copy contract AUX secretary, boss ask her off duty before hand in
hetong。
contracts
I know the secretary who will copy contracts. The boss asks her to give them to him before she leaves.

15. Wo zhidao zhege henshao zuo feiji de nüshi，ta youdian pagao。
I know this rarely take plane AUX lady, she a little afraid of heights
I know this lady who rarely takes planes. She is afraid of heights.

16. Wo zhidao zhege jingchang xie wenzhang de zuojia, henduo bianji dou xihuan tade
I know this often write article AUX author, many editor all like his

wenzhang。
article

I know this author who often writes articles. Many editors like his articles.

17. Wo renshi zhege zongshi dai yanjing de shushu, ta shi wo baba de pengyou。
I know this always wear glasses AUX uncle, he is my father AUX friend

I know this gentleman who always wears glasses. He is a friend of my father's.

18. Wo zhidao zhege jingchang qi daxiang de guowang, daxiang shi tamen guojia zui
I know this often ride eleplant AUX king, elephants is their country most

changjian de jiaotonggongju。
common AUX transportation

I know the king who often rides elephants, which is the most common transportation in his country.

19. Wo zhidao zhege changchang fa lunwen de jiaoshou, ta qunian jiu fabiaole
I know this often publish paper AUX professor, she last year ADV published

shipian lunwen。
ten pieces paper

I know this professor who often publishes papers. She published ten papers last year.

20. Wo renshi zhege zongshi du xiaoshuo de jizhe， ta shi mama de tongshi。
I know this always read novel AUX journalist, he is mother AUX colleague

I know this journalist who always reads novels. He is my mother's colleague.

21. Wo renshi zhege henshao mai duanku de zhuli， tade laoban yaoqiu tamen chuan changku
I know this rarely buy shorts AUX assistant, his boss require they wear pants

shangban。
at work

I know this assistant who rarely buys shorts, because his boss requires him to wear pants at work.

22. Wo renshi zhege congbu suan shouru de gudong, ta dui zijide gongsi tebie you
I know this never calculate income AUX shareholder, he to his company very have

xinxin。
comfident

I know this shareholder who never calculates income. He believes in his company.

23. Wo renshi zhege jingchang huan faxing de zhuchi, ta hui genju jiemu fengge
I know this often change hairstyle AUX host, she will depend on show style

xuanze butong de faxing。
choose different AUX hairstyles

I know this host who often changes hairstyle. She chooses different hairstyles depending on the style of the show.

24. Wo renshi zhege jingchang zuo dangao de chushi， wo jingchang qu tade dianli mai dangao 。
I know this often bake cake AUX chef, I sometimes go her shop buy

dangao。
cake

I know this chef who often bakes cakes, and I sometimes go to her shop to buy cakes.

25. Wo renshi zhege youshi wan shazi de xiaohai，ta shi wo linju de erzi。
I know this sometimes play sand AUX kid, he is my neighbor AUX son

I know this kid who sometimes builds sandcastles. He is my neighbor's son.

26. Wo renshi zhege ou'er kan dianying de nüren， ta geng xihuan ting jingju。
I know this sometimes see movie AUX woman, she more enjoy listen Opera

I know a woman who sometimes sees movies. She also enjoys the Peking Opera.

27. Wo renshi zhege changchang xiu diannao de yuangong， xiu diannao shi ta gongzuo
I know this usually repair computer AUX officer, repair computer is he job

de yibufen。
AUX a part

I knew this officer who usually repairs computers. Repairing computers is a part of his job.

28. Wo renshi zhege jingchang kua duishou de jiaolian， ta yong zhezhong fangfa
I know this often praise opponent AUX coach, he use this method

guli zijide duiyuan。
encourage his player

I know this coach who often praises opponents. He uses this method to encourage his players.

29. Wo renshi zhege zongshi chao baicai de gongren， ta shebude huaqian mai qitade
I know this usually cook cabbage AUX worker, he reluctant spend money buy other

cai。
vegetables

I know this worker who usually has cabbage. He is reluctant to spend money on other vegetables.

30. Wo renshi zhege changchang jian jiaozi de geshou， ta mei taiduo shijian zuo qita
I know this often cook dumpling AUX singer, he doesn't much time cook other

chide。
food

I know a singer who often cooks dumplings. He doesn't have enough time to cook other dishes.

31. Wo zhidao zhege henshao reng wanju de ying’er, ta shi wo jianguo de
I know this seldom throw toy AUX baby, she is my have seen AUX

zuiguaide ying’er。
most well-behaved baby

I know this baby who seldom throws toys. She is the most well-behaved baby I have ever seen.

32. Wo renshi zhege henshao chi jidan de shaonian, ta chi jidan hui laduzi。
I know this seldom eat egg AUX teenager, he eat egg will have diarrhea

I know this teenager who seldom eats eggs. He will have diarrhea if he eats eggs.

**1.2 Original sentences in Ex2**

1. Wo ting Xiaotao shuo nage changchang fa lunwen de jiaoshou jinnian you
I heard from Xiaotao say the always publish paper AUX professor this year again

fabiaole shipian lunwen，ta tai lihai le。
published ten pieces paper, he too great AUX

I heard from Jay that the professor who always publishes papers published ten more this year.

2. Wo ting Xiaoyu shuo nage yizhi zu huabi de sheji meiyou guding de
I heard from Xiaoyu say that always rent brush AUX designer have no fixed AUX

shouru，ta maibuqi haode huabi。
income, she can't afford good brushes.

I heard from Tom that the designer who always rents brushes has no fixed income, and she can't afford good brushes.

3. Wo ting jiejie shuo nage zongshi jiang xiaohua de jieshuo canjiale jintian de
I heard from sister say that always tell joke AUX commentator joined today AUX

zhibo，tade zhibo yiding hen youyisi。
live, his show must very interesting.

I heard from my sister that the commentator who always tells jokes will give a live show. His show must be interesting.

4. Wo ting Tiantian shuo nage congbu suan shouru de gudong pochanle，ta dui
I heard from Tiantian say that never calculate income AUX shareholder bankrupt, he to

gongsi taiguo zixin le。
company too confident AUX

I heard from Lucy that the shareholder who never calculates income went bankrupt. He was too confident.

5. Wo ting bianji shuo nage jingchang xie wenzhang de zuojia shoudaole haoduo duzhe
I heard from editor say that often write article AUX author received many reader

laixin，tade wenzhang tai huo le。
letters, his article too popular AUX

I heard from the editor that the author who often writes articles receives many letters from readers.

6. Wo ting Xiao'ai shuo nage congbu yong rili de zhuxi you wangji kaihui
I heard from Xiao'ai say that never check calender AUX president again forget meeting

le， henduo ren dou jianyi ta yong rili tixing。
AUX, manyo people all suggest him use calender reminder

I heard from Mary that the president who never checks calendars forgot the meeting. He needs someone to remind him.

7. Wo ting Longlong shuo nage yizhi yang xiaogou de a'yi you yangle yizhi
I heard from Longlong say that always raise puppy AUX woman again raised one

xiaomao，ta tebie xihuan xiaodongwu。
kitten, she very like pet

I heard from John that the woman who always raises puppies raised a kitten. She likes pets very much.

8. Wo ting Xiaomei shuo nage changchang bei chengyu de laowai yijing biyele，
I heard from Xiaomei say that always recite idiom AUX foreigner already graduated,

guaibude henjiu mei jiandao ta le。
no wonder long time not see him AUX

I heard from Lucy that the foreigner who always recites idioms has graduated. No wonder I haven't seen him recently.

9. Wo ting Weiwei shuo nage jingchang da wangqiu de jingli you dele diyiming，
I heard from Weiwei say that often play tennis AUX manager again won first prize,

ta qunian jiushi diyiming。
he last year is first prize

I heard from Lily that the manager who often plays tennis got the first prize again, as he did last year.

10. Wo ting Xiaoli shuo nage congbu ma xiashu de laoban cizhile，tade yuangong
I heard from Xiaoli say that never scold employee AUX boss resigned, his employee

dou feichang shebude ta。
all very miss him

I heard from Mary that the boss who never scolded employees resigned. His employees missed him.

11. Wo ting Xiaoxiao shuo nage zongshi yao shouzhi de ying'er qu kan yisheng le， ta
I heard from Xiaoxiao say that always bite finger AUX baby go see doctor AUX, his

fumu juede ta keneng queshao yingyang。
parents think he may lack nutrition

I heard from Helen that the baby who always bites fingers went to the hospital. His parents think he may lack nutrition.

12. Wo ting Xixi shuo nage jingchang kan huaju de qingnian kaoshangle huajuyuan，ta
I heard from Xixi say that often see opera AUX guy enrolled in theatre, he

hui chengwei yiming youxiude yanyuan。
will be a good actor

I heard from Lin that the guy who often sees movies was enrolled in a theatre. He will be a good actor.

13. Wo ting Dawei shuo nage jingchang jie yusan de guke conglaibu kan
I heard from Dawei say that always borrow umbrella AUX customer never watch

tianqiyubao， meici xiayu ta doubu dai san。
weather report, every time rain he never bring umbrella

I heard from Tommy that the customer who always borrows umbrellas never watches the weather report.

14. Wo ting Danan shuo nage jingchang song liwu de caifeng he nanpengyou
I heard from Danan say that always give present AUX dressmaker with boyfriend

fenshoule，ta yiding tebie shangxin。
broke up, she must very sad

I heard from Lay that the dressmaker who always gave presents broke up with her boyfriend. She must be very sad.

15. Wo ting Meimei shuo nage jingchang chang minyao de nanren bei fensi
I heard from Meimei say that often sing ballad AUX man AUX fans

tuantuanweizhu，ta you henduo fensi。
surrounded, he have a lot of fans

I heard from Lisa that the man who often sings ballads is surrounded by fans. He has a lot of fans.

16. Wo ting Xixi shuo nage jingchang chou xiangyan de xiaotou bei zhuaqilaile，ta
I heard from Xixi say that often smoke cigarette AUX thief AUX was arrested, he

zai maiyan de shihou bei faxianle。
AUX buy cigarette AUX time AUX found

I heard from Amy that the thief who often smoked cigarettes was arrested. He was caught when buying cigarettes.

17. Wo ting bianji shuo nage jingchang du xiaoshuo de jizhe beikaichule，ta zai
I heard from editor say that always read novel AUX journalist was fired, he at

gongzuozhong fanle taiduo cuowu。
in work made much mistake

I heard from Helen that the journalist who always read novels was fired. He made too many mistakes.

18. Wo ting Meimei shuo nage congbu chi jidan de shaonian shengbingle，yisheng rang
I heard from Meimei say that never eat egg AUX teenager was sick, doctor ask

ta duo hedian niunai。
him more drink milk

I heard from Tina that the teenager who never ate eggs was sick. The doctor told him to drink more milk.

19. Wo ting Xiaoxiao shuo nage zongshi si keben de nanhai zheci kaoshi you
I heard from Xiaoxiao say that always tear textbook AUX boy this time exam again

bujige，ta xuexi tebie bu yongxin。
fail, he study very not hard

I heard from Vicky that the boy who always tore textbooks failed the exam again. He didn't study hard.

20. Wo ting laoban shuo nage jingchang jie dianhua de zhuli cizhile，ta bu
I heard from boss say that often answer phone AUX secretary resigned, she didn't

xihuan zheme wuliaode gongzuo。
like such boring work

I heard from the boss that the secretary who often answered phones resigned. She didn't like such boring work.

21. Wo ting Xiaomei shuo nage jingchang hua ditu de daoyou dang laoban le，ta
I heard from Xiaomei say that often draw map AUX guide become boss AUX, he

xianzai zhuyao mai ziji hua de ditu。
now mainly sell self draw AUX map

I heard from Ann that the guide who often draws maps became a boss. Now he sells the maps he draws.

22. Wo ting Xiaowei shuo nage jingchang jiao gushi de zhuanjia yao tuixiu le，wo
I heard from Xiaowei say that often teach poetry AUX expert will retire AUX, I

dasuan zai quting yici tade ke。
plan to again attend once her class

I heard from James that the expert who often teaches poetry is retiring. I want to attend her class again.

23. Wo ting baba shuo nage zongshi dai yanjing de shushu xiazhou yao lai
I heard from father say that always wear glasses AUX gentleman next week will come

wojia， he women yiqi chifan。
my house, with us together have dinner

I heard from my father that the gentleman who always wears glasses plans to come to my house next week.

24. Wo ting Dazhuang shuo nage henshao zuo feiji de nüshi zuo huoche qu faguo
I heard from Dazhuang say that rarely take plane AUX lady take train go to France

le， ta zhende feichang konggao。
AUX, she really very afraid of heights

I heard from Roy that the lady who rarely takes planes went to France by train. She is afraid of heights.

25. Wo ting Xiaobo shuo nage congbu qing fanyi de lingdao hui shuo henduo guojia
I heard from Xiaobo say that never use interpreter AUX leader can speak many country

de yuyan，ta zhen lihai。
AUX language, he too great

I heard from Jorge that this leader who never uses interpreters speaks many different languages.

26. Wo ting Dali shuo nage dasuan xue fayu de xuesheng baole wanshang de
I heard from Dali say that plan to study French AUX student signed up evening AUX

ke， ta xue fayu shiweile qu liuxue。
class, she study French is for go to study abroad

I heard from Tom that the student who will study French signed up for the evening class. She studies for studying abroad.

27. Wo ting Lulu shuo nage jingchang juan jiangjin de guanjun zheci you ba
I heard from Lulu say that often donate prizes AUX champion this time again AUX

jiangjin juangeile cishan jigou。
reward donated charty organization

I heard from Nancy that the champion who often donated prizes won a national reward and donated it again.

28. Wo ting Lingling shuo nage changchang xiu diannao de yuangong huan gongzuo
I heard from Lingling say that usually reapair computer AUX officer change job

le， ta qule yijia diannao gongsi。
AUX, he went a computer company

I heard from Wendy that the officer who usually repairs computers changed his job and went to a computer company.

29. Wo ting duiyuan shuo nage jingchang kua duishou de jiaolian likai duiwu le，
I heard from player say that always praise opponent AUX coach leave team AUX,

xinde jiaolian haimei shangren。
new coach hasn't been appointed.

I heard from the players that the coach who always praised opponents left the team. The new coach hasn't been appointed.

30. Wo ting Tingting shuo nage jingchang jian jiaozi de bao'an qu chaoshi
I heard from Tingting say that often fry dumpling AUX guard go to supermarket

le， chaoshi de jiaozi zai dazhe。
AUX, supermarket AUX dumpling is on sale

I heard from Rose that the guard who often fried dumplings went to the supermarket, where the dumplings were on sale.

31. Wo ting Xiaopeng shuo nage changchang zhu yumi de linju banjiale， ta
I heard from Xiaopeng say that always boil corn AUX neighbor moved away, she

zhiqian song yumi gei women chi。
before give corn to us eat

I heard from Allen that the neighbor who always boiled corns moved away. She used to give us corn to eat.

32. Wo ting Xiaowen shuo nage zongshi ban guizi de gongren hui laojia le，
I heard from Xiaowen say that always move cabinet AUX worker back hometown AUX,

tade muqin shengbing le。
his mother ill AUX

I heard from Wendy that the worker who always moves cabinets has gone back to his hometown. His mother is ill.

# 2 Supplementary Figures and Tables

## 2.1 Supplementary Figures in ex1

**Supplementary Figure 1**: linear mixed model results in det area

| | Fixed effects | | | | | Random effects | |
|---|---|---|---|---|---|---|---|
| | **Estimated SD** | **Error** | ***t*** | ***p*** | | **variation** | **error** |
| **Intercept** | 5.88 | 0.06 | 103.87 | | **Item** | 0.00 | 0.03 |
| **Lan** | 0.07 | 0.03 | 2.24 | 0.025* | **Subject** | 0.07 | 0.26 |
| **Con** | -0.02 | 0.03 | -0.75 | 0.457 | | | |
| **Lan * con** | 0.03 | 0.04 | 0.61 | 0.541 | | | |

Note: Lan-language, con-consistency;
The model is log(rt)~lan + con + lan:con + (1|subject)+(1|item).

**Supplementary Figure 2**: linear mixed model results in V area

| | Fixed effects | | | | | Random effects | |
|---|---|---|---|---|---|---|---|
| | **Estimated SD** | **Error** | ***t*** | ***p*** | | **variation** | **error** |
| **Intercept** | 5.96 | 0.06 | 99.86 | | **Item** | 0.00 | 0.04 |
| **Lan** | 0.02 | 0.03 | 7.43 | 0.000*** | **Subject** | 0.08 | 0.28 |
| **Con** | -0.05 | 0.03 | -1.55 | 0.121 | | | |
| **Lan * con** | 0.03 | 0.05 | 0.70 | 0.484 | | | |

Note: Lan-language, con-consistency;
The model is log(rt)~lan + con + lan:con + (1|subject)+(1|item).

**Supplementary Figure 3**: linear mixed model results in Inner N area

| | Fixed effects | | | | | Random effects | |
|---|---|---|---|---|---|---|---|
| | **Estimated SD** | **Error** | ***t*** | ***p*** | | **variation** | **error** |
| **Intercept** | 6.21 | 0.08 | 79.39 | | **Item** | 0.00 | 0.06 |
| **Lan** | 0.01 | 0.04 | 2.58 | 0.010** | **Subject** | 0.13 | 0.36 |
| **Con** | 0.02 | 0.04 | 0.66 | 0.510 | | | |
| **Lan * con** | -0.01 | 0.06 | -2.44 | 0.015* | | | |

Note: Lan-language, con-consistency;
The model is log(rt)~lan + con + lan:con + (1|subject)+(1|item).

**Supplementary Figure 4**: linear mixed model results in Outer N area

| | Fixed effects | | | | | Random effects | |
|---|---|---|---|---|---|---|---|
| | **Estimated SD** | **Error** | ***t*** | ***p*** | | **variation** | **error** |
| **Intercept** | 6.06 | 0.07 | 92.34 | | **Item** | 0.00 | 0.00 |
| **Lan** | 0.08 | 0.04 | 2.36 | 0.019* | **Subject** | 0.10 | 0.31 |
| **Con** | -0.06 | 0.03 | -1.71 | $0.088^{-}$ | | | |
| **Lan * con** | -0.10 | 0.05 | 2.07 | 0.039* | | | |

Note: Lan-language, con-consistency;

The model is log(rt)~lan + con + lan:con + (1|subject)+(1|item).

## 2.2 Supplementary Figures in ex2

**Supplementary Figure 5**: linear mixed model results in Det area

| | Fixed effects | | | | | Random effects | |
|---|---|---|---|---|---|---|---|
| | **Estimated SD** | **Error** | ***t*** | ***p*** | | **variation** | **error** |
| **Intercept** | 6.06 | 0.07 | 92.34 | | **Item** | 0.00 | 0.03 |
| **Lan** | 0.08 | 0.04 | 2.36 | 0.019* | **Subject** | 0.09 | 0.30 |
| **Con** | -0.06 | 0.03 | -1.71 | $0.088^{-}$ | | | |
| **Back** | -0.08 | 0.11 | -0.76 | 0.449 | | | |
| **Lan * Con** | 0.09 | 0.05 | 1.93 | $0.054^{-}$ | | | |
| **Lan* Back** | -0.08 | 0.05 | -1.55 | 0.122 | | | |
| **Con * Back** | 0.02 | 0.05 | 0.31 | 0.756 | | | |
| **Lan*Con*Back** | -0.09 | 0.07 | -1.28 | 0.202 | | | |

Note: Lan-language, con-consistency, Back-language background;

The model is log(rt)~lan + con + back + lan:con + lan:back + back:con+ lan:back:con + (1|subject)+(1|item).

**Supplementary Figure 6**: linear mixed model results in V area

| | Fixed effects | | | | | Random effects | |
|---|---|---|---|---|---|---|---|
| | **Estimated SD** | **Error** | ***t*** | ***p*** | | **variation** | **error** |
| **Intercept** | 5.91 | 0.07 | 84.94 | | **Item** | 0.00 | 0.04 |
| **Lan** | 0.21 | 0.04 | 5.58 | 0.000*** | **Subject** | 0.09 | 0.29 |
| **Con** | 0.05 | 0.04 | 1.39 | 0.166 | | | |
| **Back** | -0.04 | 0.11 | -0.41 | 0.680 | | | |
| **Lan * Con** | -0.02 | 0.05 | -0.35 | 0.726 | | | |
| **Lan* Back** | -0.27 | 0.06 | -4.78 | 0.000*** | | | |
| **Con * Back** | -0.08 | 0.06 | -1.43 | 0.153 | | | |
| **Lan*Con*Back** | 0.04 | 0.06 | 0.45 | 0.650 | | | |

Note: Lan-language, con-consistency, Back-language background;

The model is log(rt)~lan + con + back + lan:con + lan:back + back:con+ lan:back:con + (1|subject)+(1|item).

**Supplementary Figure 7**: linear mixed model results in Inner N area

| | Fixed effects | | | | | Random effects | |
|---|---|---|---|---|---|---|---|
| | **Estimated SD** | **Error** | ***t*** | ***p*** | | **variation** | **error** |
| **Intercept** | 6.19 | 0.09 | 71.87 | | **Item** | 0.00 | 0.05 |

| | | | | | | | |
|---|---|---|---|---|---|---|---|
| **Lan** | 0.04 | 0.05 | 0.99 | 0.321 | **Subject** | 0.13 | 0.36 |
| **Con** | 0.00 | 0.05 | 0.11 | 0.910 | | | |
| **Back** | -0.19 | 0.13 | -1.47 | 0.141 | | | |
| **Lan * Con** | 0.07 | 0.06 | 1.16 | 0.246 | | | |
| **Lan* Back** | -0.08 | 0.07 | -1.13 | 0.260 | | | |
| **Con * Back** | -0.01 | 0.07 | -0.18 | 0.854 | | | |
| **Lan*Con*Back** | 0.05 | 0.10 | 0.52 | 0.604 | | | |

Note: Lan-language, con-consistency, Back-language background;
The model is log(rt)~lan + con + back + lan:con + lan:back + back:con+ lan:back:con + (1|subject)+(1|item).

**Supplementary Figure 8**: linear mixed model results in Outer N area

| | **Fixed effects** | | | | | **Random effects** | |
|---|---|---|---|---|---|---|---|
| | **Estimated SD** | **Error** | ***t*** | ***p*** | | **variation** | **error** |
| **Intercept** | 5.96 | 0.08 | 73.98 | | **Item** | 0.01 | 0.08 |
| **Lan** | 0.20 | 0.04 | 4.71 | 0.000*** | **Subject** | 0.11 | 0.34 |
| **Con** | -0.07 | 0.04 | -1.67 | $0.096^{-}$ | | | |
| **Back** | -0.02 | 0.12 | -0.14 | 0.886 | | | |
| **Lan * Con** | 0.12 | 0.06 | 2.00 | 0.046* | | | |
| **Lan* Back** | -0.29 | 0.06 | -4.67 | 0.000*** | | | |
| **Con * Back** | 0.04 | 0.06 | 0.70 | 0.487 | | | |
| **Lan*Con*Back** | 0.03 | 0.09 | 0.31 | 0.758 | | | |

Note: Lan-language, con-consistency, Back-language background;
The model is log(rt)~lan + con + back + lan:con + lan:back + back:con+ lan:back:con + (1|subject)+(1|item).

**Supplementary Figure 9**: linear mixed model results in the whole RC area

| | **Fixed effects** | | | | | **Random effects** | |
|---|---|---|---|---|---|---|---|
| | **Estimated SD** | **Error** | ***t*** | ***p*** | | **variation** | **error** |
| **Intercept** | 7.50 | 0.09 | 84.26 | | **Item** | 0.00 | 0.06 |
| **Lan** | 0.19 | 0.04 | 5.35 | 0.000*** | **Subject** | 0.15 | 0.39 |
| **Con** | 0.00 | 0.03 | 0.13 | 0.894 | | | |
| **Back** | 0.14 | 0.13 | 1.06 | 0.288 | | | |
| **Lan * Con** | 0.08 | 0.05 | 1.56 | 0.119 | | | |
| **Lan* Back** | -0.28 | 0.05 | -5.15 | 0.000*** | | | |
| **Con * Back** | -0.00 | 0.05 | -0.08 | 0.935 | | | |
| **Lan*Con*Back** | 0.02 | 0.08 | 0.27 | 0.786 | | | |

Note: Lan-language, con-consistency, Back-language background;
The model is log(rt)~lan + con + back + lan:con + lan:back + back:con+ lan:back:con + (1|subject)+(1|item).
